# Supplementary material for: Alterations in the Epigenetic Machinery Associated with Prostate Cancer Health Disparities
Source: Cancers (Basel). 2023 Jul 1;15(13):3462. doi: 10.3390/cancers15133462 (PMC10341375; doi:10.3390/cancers15133462)
Supplement: Supplementary file 1 [file cancers-15-03462-s001.zip › Cancers_Suppl_Figures.pdf]

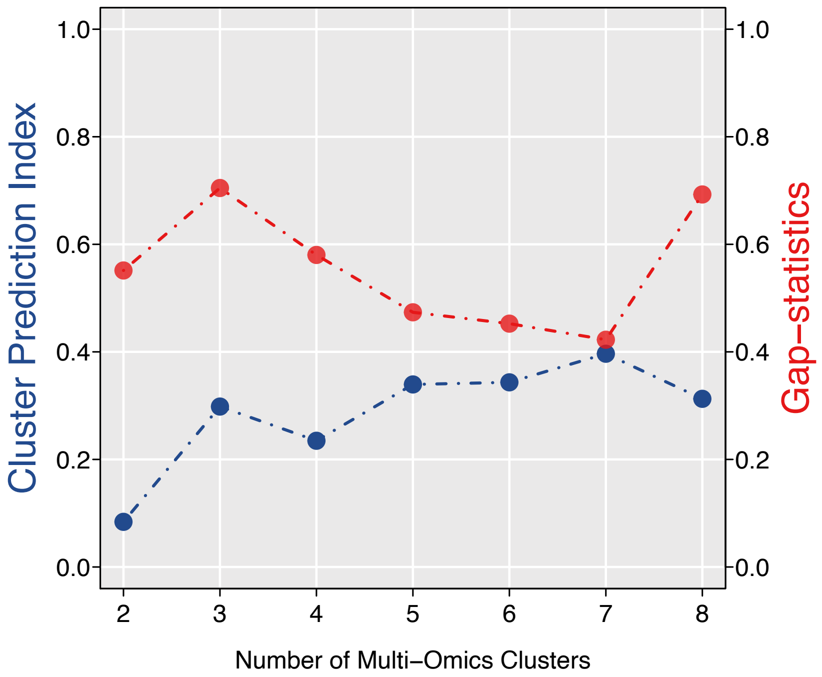

Figure S1. Optimal cluster number identification. Using the MOVICS (Multi-Omics integration and VIsualization in Cancer Subtyping) R package, identification of the optimal cluster number for variant data by calculating Cluster Prediction Index (CPI, blue line) and Gap statistic (red line), reveals 8 and 3 as potentially optimal cluster numbers.

A

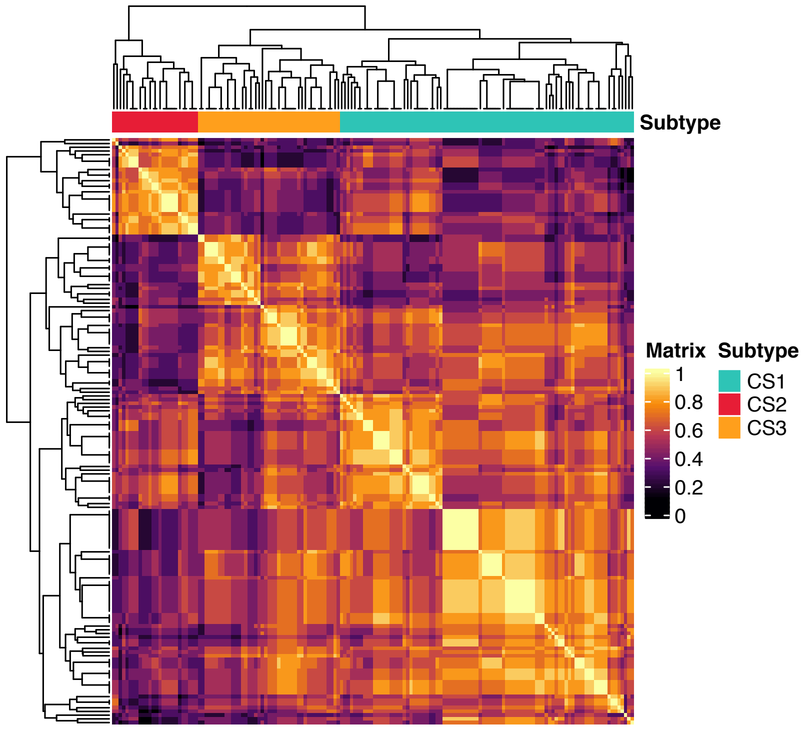

B

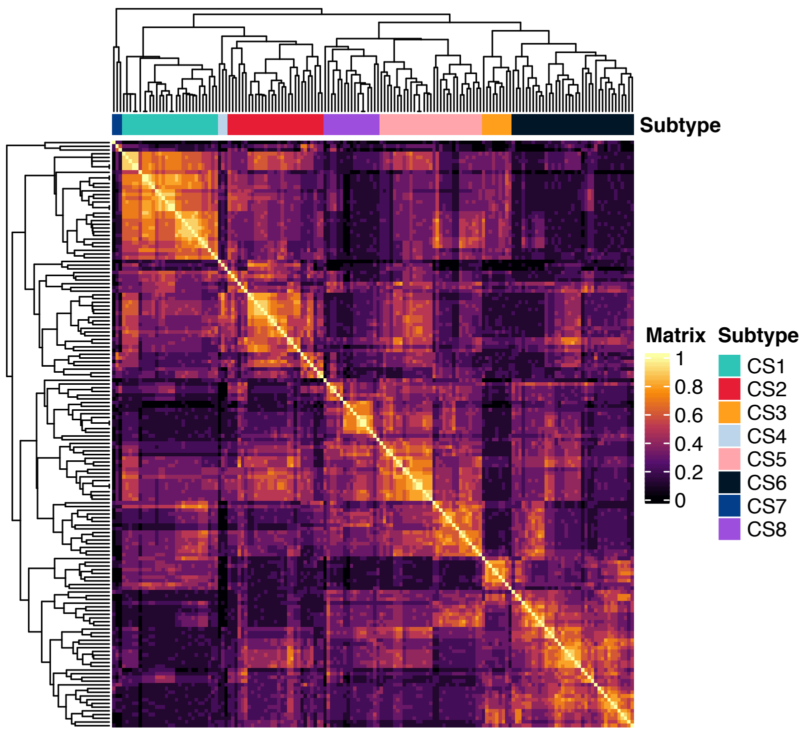

Figure S2. Consensus heatmap for variant data overlapping epigenetic machinery genes based on results from ten multi-omics integrative clustering algorithms with the assigned cluster number of (A) k = 3 and (B) k = 8. The consensus matrix is a probability matrix that details how many times samples belonging to a subtype would be clustered together again by different clustering methods. We found k = 3 best clusters the variant data into distinct subtypes.

A

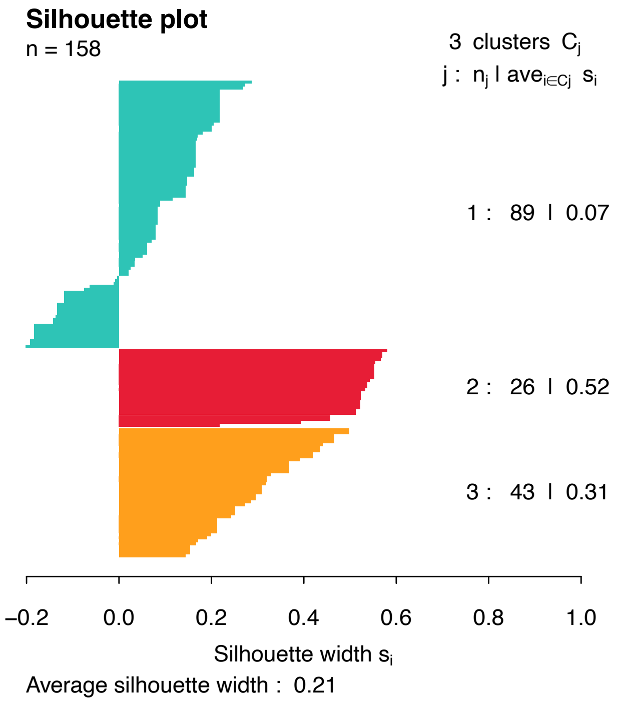

B

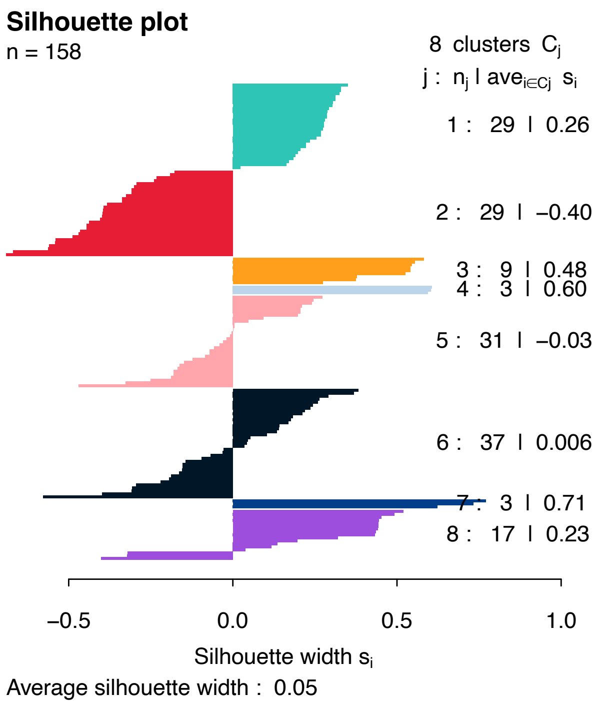

Figure S3. Silhouette plot quantifying sample similarity based on results from ten multi-omics integrative clustering algorithms with the assigned cluster number of (A) k = 3 and (B) k = 8. An average silhouette coefficient closer to +1 indicates that samples have been assigned to distinctly different clusters i.e. samples are far away from the neighboring clusters. Conversely, negative values indicate samples have likely been assigned to the wrong cluster. Evidently, k = 3 shows a higher average silhouette coefficient, with fewer samples displaying negative coefficients, in comparison to k = 8.

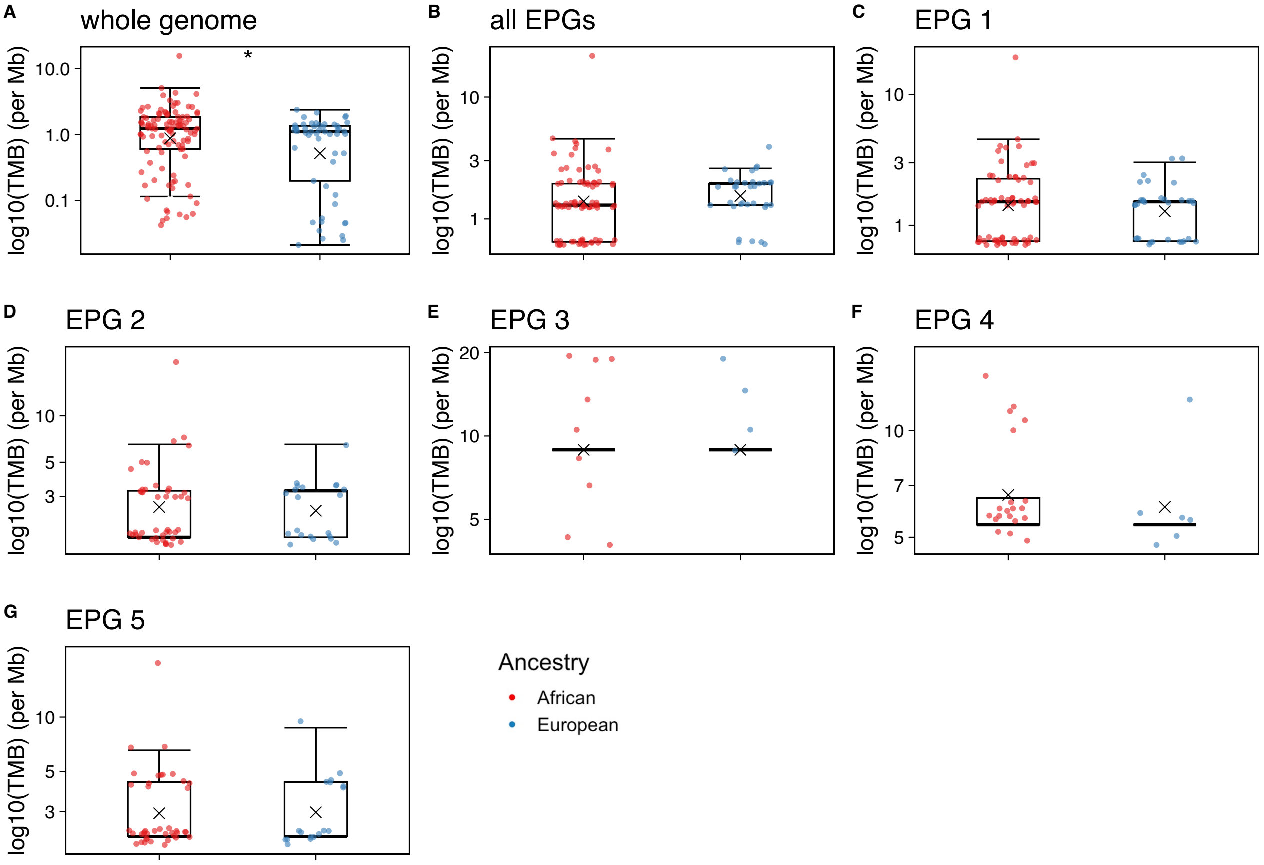

Figure S4. Mutational burden in African and European derived tumors. The box plots show the median (center line), the mean (cross), the 25th and 75th percentiles (box limits), and ±1.5× the interquartile range (whiskers). (A) For each sample, tumor mutational burden is shown for the whole genome (n = 105 Africans [mean = 1.47, median = 1.23, range = 0.042-15.7] and n = 53 Europeans [mean = 0.94, median = 1.10, range = 0.021-2.38]). For each sample, total somatic coding variants (SNVs and indels) divided by total coding base pairs for each respective group is shown for (B) epigenetic process groups 1-5 combined (n = 77 Africans [mean = 1.84, median = 1.30, range = 0.649-21.4] and n = 33 Europeans [mean = 1.69, median = 1.95, range = 0.649-3.90]); (C) epigenetic process group 1 (n = 66 Africans [mean = 1.87, median = 1.51, range = 0.756-19.7] and n = 32 Europeans [mean = 1.42, median = 1.51, range = 0.756-3.02]); (D) epigenetic process group 2 (n = 44 Africans [mean = 3.23, median = 1.63, range = 1.63-24.5] and n = 21 Europeans [mean = 2.64, median = 3.26, range = 1.63-6.52]); (E) epigenetic process group 3 (n = 9 Africans [mean = 8.91, median = 8.91, range = 8.91-8.91] and n = 4 Europeans [mean = 8.91, median = 8.91, range = 8.91-8.91]); (F) epigenetic process group 4 (n = 20 Africans [mean = 7.05, median = 5.42, range = 5.42-16.3] and n = 6 Europeans [mean = 6.33, median = 5.42, range = 5.42-10.8]); (G) epigenetic process group 5 (n = 41 Africans [mean = 3.46, median = 2.18, range = 2.18-21.8] and n = 18 Europeans [mean = 3.28, median = 2.18, range = 2.18-8.73]). EPG: epigenetic process group; Mb: million base pairs; TMB: tumor mutational burden; *: p-value < 0.05.

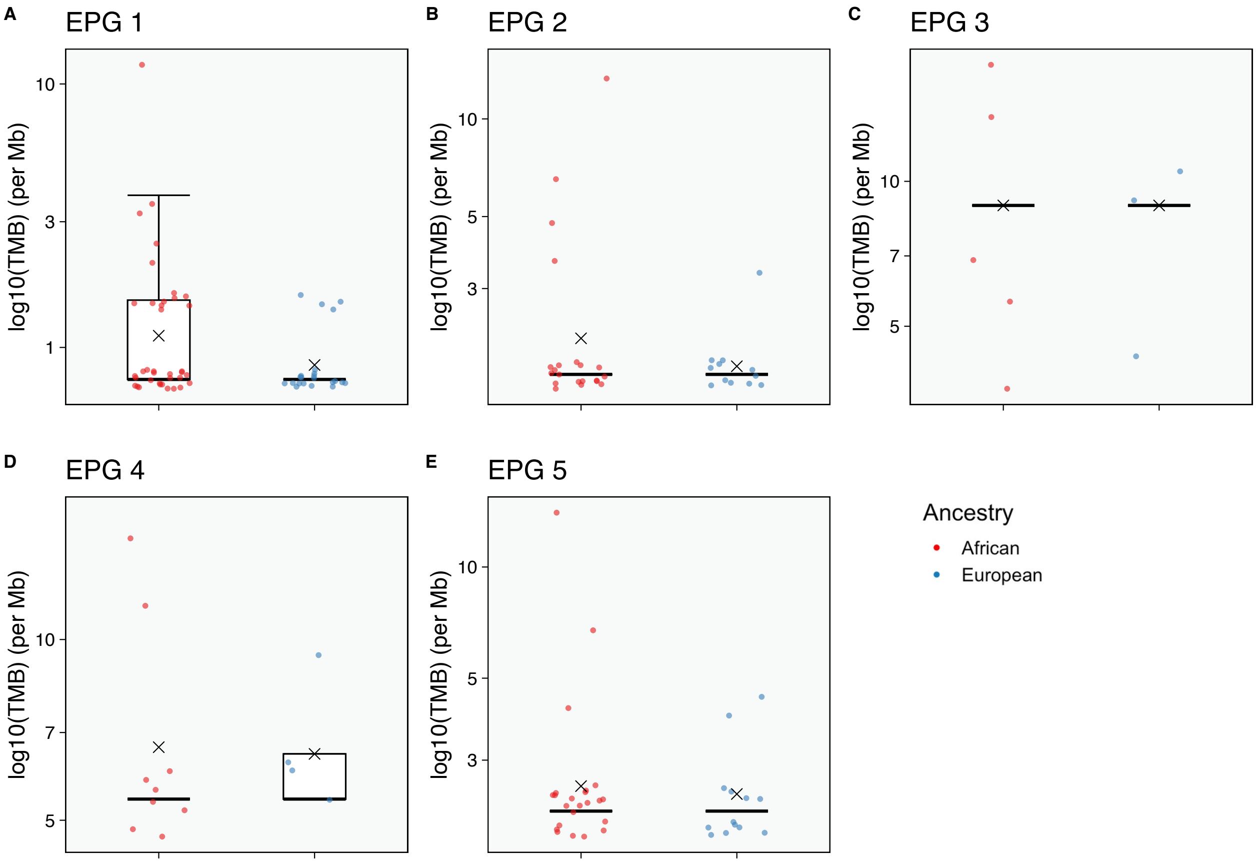

Figure S5. Damaging variant mutational burden in African and European derived tumors. The box plots show the median (center line), the mean (cross), the 25th and 75th percentiles (box limits), and ±1.5× the interquartile range (whiskers). For each sample, total potentially damaging variants, as per functional impact prediction, divided by total coding base pairs for each respective group is shown for (A) epigenetic process group 1 (*n* = 37 Africans [mean = 1.45, median = 0.756, range = 0.756-11.3] and *n* = 22 Europeans [mean = 0.89, median = 0.756, range = 0.756-1.51]); (B) epigenetic process group 2 (*n* = 21 Africans [mean = 2.72, median = 1.63, range = 1.63-14.7] and *n* = 12 Europeans [mean = 1.77, median = 1.63, range = 1.63-3.26]); (C) epigenetic process group 3 (*n* = 5 Africans [mean = 8.91, median = 8.91, range = 8.91-8.91] and *n* = 3 Europeans [mean = 8.91, median = 8.91, range = 8.91-8.91]); (D) epigenetic process group 4 (*n* = 9 Africans [mean = 7.23, median = 5.42, range = 5.42-16.3] and *n* = 4 Europeans [mean = 6.78, median = 5.42, range = 5.42-10.8]); (E) epigenetic process group 5 (*n* = 23 Africans [mean = 2.94, median = 2.18, range = 2.18-13.1] and *n* = 13 Europeans [mean = 2.52, median = 2.18, range = 2.18-4.37]). EPG: epigenetic process group; Mb: million base pairs; TMB: tumor mutational burden.
